# What are the current diabetic foot assessment methods in private podiatry practices in Flanders, Belgium: an exploratory mixed method study

**DOI:** 10.1186/s13047-023-00615-1

**Published:** 2023-03-27

**Authors:** Irene Vansteenland, Rachel Forss

**Affiliations:** 1 School of Sport & Health Sciences, University of Brighton, 49 Darley Road, Eastbourne, BN20 7UR UK; 2 Podoconsult Vansteenland Irene BV, Hendrik Consciencelaan 54, 9950 Lievegem, Belgium; 3 Centre for Regenerative Medicine and Devices, University of Brighton, Brighton, UK; 4 School of Sport and Health Sciences, University of Brighton, 49 Darley Road, Eastbourne, BN20 7UR UK

**Keywords:** Diabetes Mellitus, Diabetic Foot, Guideline, Qualitative Research, Surveys and Questionnaires, Interview, Podiatry, Belgium

## Abstract

**Background:**

Diabetic foot assessments detect patients at risk for developing a diabetes-related foot ulceration and can significantly reduce the risk of amputation. In order to organize this assessment effectively, diabetic foot assessment guidelines are required according to the International Working Group of the Diabetic Foot. However, these international guidelines have not been adapted into a national guideline for podiatrists in Flanders, Belgium. This study aims to identify the methods and guidelines currently used to assess the diabetic foot in private podiatry practices in Flanders, Belgium and to explore the podiatrists’ opinions on developing a national diabetic foot assessment guideline.

**Methods:**

This exploratory mixed method study was composed of an anonymous online survey comprising of open- and closed-ended questions followed by 1:1 online semi-structured interviews. Participants were recruited via e-mail and a closed private Facebook group of podiatry alumni. Data was analyzed using SPSS statistics and thematic analysis described by Braun and Clarke.

**Results:**

This study showed that the vascular assessment of the diabetic foot exists solely of a medical history and palpation of the pedal pulses. Non-invasive tests such as doppler, toe brachial pressure index or ankle brachial pressure index are seldom used. Only 66% reported to use a guideline for the diabetic foot assessment. There was a variety of reported guidelines and risk stratification systems in use in private podiatry practices in Flanders, Belgium.

**Conclusion:**

Non-invasive tests such as the doppler, ankle brachial pressure index or toe brachial pressure index are rarely used for the vascular assessment of the diabetic foot. Diabetic foot assessment guidelines and risk stratification systems to identify patients at risk for developing a diabetic foot ulcer were not frequently used. International guidelines of the International Working Group of the Diabetic Foot have not yet been implemented in private podiatry practices in Flanders, Belgium. This exploratory research has provided useful information for future research studies.

## Background

The prevalence of diabetes mellitus (DM) is increasing at an alarming rate. In 2018, the prevalence of diabetes in the Belgian population has increased to 6.1% as a result of the population ageing and an increase in overweight or obesity [1]. Diabetes-related foot ulceration (DFU) is one of the most prevalent and serious complications of DM [2]. The annual incidence of DFUs in Belgium is 2% and the lifetime risk of developing a DFU has been estimated between 19 and 34% [3, 4]. Moreover, recent data suggest that between 25 to 40% of patients with a history of a DFU experience a recurrence within 1 year after the ulcer has healed [3, 4]. DFUs are the most common cause of non-traumatic amputation in Western countries, with 85% of all lower limb amputations reported as being preceded by a DFU [5]. Early detection of the patients at risk for developing a DFU, through a diabetic foot assessment, can significantly reduce the risk of amputation [6, 7]. The study of Lavery et al. showed that implementation of a diabetic foot assessment with complementary preventive and acute care services, according to patient’s risk factor, reduces the incidence of amputations by 47% [7]. Peer reviews of the diabetic foot services in the South-West region of the UK indicated that introducing regular foot examinations and offering advice or referral to preventive and acute services decreases the incidence of DFU related major amputations [8]. Podiatrists have an essential role in performing these foot assessments and providing preventive or acute services. The study of Blanchette et al. [9] reported that multidisciplinary teams with podiatry services lead to a significant reduction in lower extremity amputations. Moreover, providing podiatry services for patients with diabetes before the onset of a DFU reduces hospital admissions [10]. In Belgium, the initial diabetic foot assessment is carried out by the general practitioner (GP). When patients are at low or moderate risk of developing a DFU, they are referred to podiatrists, working in the private sector, for annual diabetic foot assessments and prevention services [11]. In order to organize these assessments and services effectively, guidelines are required according to the International Working Group of the Diabetic Foot (IWGDF) [12]. The IWGDF has developed an evidence-based international diabetic foot assessment guideline for all health care professionals [13, 14]. This practical guideline is aimed at the global community of health care professionals involved in the diabetic foot care [14]. The working group recommend that those guidelines may have to be adapted based on local circumstances taking into account accessibility to health care resources and various cultural factors [14]. However, to date, these international diabetic foot assessment guidelines have not been adapted into a national guideline for podiatrists working in the private sector in Flanders, Belgium. Therefore, the primary objective of this research was to examine which methods and guidelines are currently used to assess the diabetic foot in private podiatry practices in Flanders, Belgium. The second objective of this study was to explore the podiatrists’ perceptions on developing a national guideline for the diabetic foot assessment in Flanders, Belgium.

## Methods

To our knowledge, this is the first research investigating the diabetic foot assessment methods in private podiatry practices in Flanders, Belgium. Therefore, an exploratory mixed method research was conducted. The quantitative phase of this research involved the collection of data by using an anonymous online survey to determine which methods and guidelines are currently used to assess the diabetic foot. A sequential qualitative phase followed the quantitative phase to clarify the results of the survey an to explore podiatrists’ perceptions on developing a national diabetic foot assessment guideline. This phase consisted of online 1:1 interviews. The target population of this study were podiatrists registered with the Belgian National Institute for Health and Disability Insurance (NIHDI) and working in the private sector in Flanders, Belgium. The School of Health Sciences Research Ethics Panel, University of Brighton approved the study on the 11^th^ of March 2021.

### Study design

An anonymous online survey comprising of 6 open- and 8 closed-ended questions was drawn up to generate quantitative data. These questions were generated following a literature review. The IT services of the Jisc UK digital, data and technology agency were used to host the online survey. The front page of the survey included information on storage of data, purpose of the study and data protection. Participants had to give their informed consent in order to gain access to this survey. The survey questions were distributed on 2 screen pages and were translated in English by a translator. Participants could choose to write the answers to the open-ended questions in their preferred language to encourage participation. They could review and change their answers before finishing the survey. All questions had to be completed in order to finish the survey. A pilot of this survey was completed by the researcher, supervisor and an experienced podiatrist, prior to sending out the survey links.

From May until July 2021, invitations to participate in the online survey were sent via e-mail to 362 podiatrists working in the private sector in Flanders, Belgium. These e-mail addresses were retrieved from an internet search and were publicly available. The survey was also promoted on the private Facebook group of all podiatry alumni of the Artevelde University in Flanders, Belgium. Reminder e-mails and Facebook posts were sent two weeks after the initial invitation or post to improve the response rate. In order to retrieve qualitative data on podiatrist’s perceptions on developing a national guideline, survey respondents were asked in the survey to indicate if they were interested in participating in a one-on-one semi-structured interview. Only 9 out of the 50 participants indicated they would like to participate in these interviews. Invitations for these interviews were sent via e-mail in July 2021. After the invitation e-mails were sent out, only 4 volunteers were interested to take part in the next step of this research.

The researcher conducted the interviews in Dutch. These interviews were held online and recorded via Microsoft Teams. The interviews recordings were deleted after transcription. The interviews lasted approximately 1 h and were carried out once per participant, repeat interviews were not conducted. The researcher developed an interview guide which outlined the structure of the interview and was used as a tool to check if every question was answered by the interviewees. This guide was reviewed by the supervisor prior to the first interview. The interviews were coded using thematic inductive analysis described by Braun and Clarke [15]. The 3 core themes developed from this analysis are presented in Table 1 and the identified subthemes are also illustrated. Participating in the survey and interviews was voluntary. There were no incentives or rewards offered to participate in this research.

**Table 1:** Themes thematic analysis

Themes	Subthemes
Guidance documents or guidelines for the diabetic foot assessment	-Use of guidelines in private podiatry practices -Retrieving guidelines -Communication of the latest international guidelines
Diabetic foot assessment	-Use of a Doppler -Inconsistencies in the interpretation of the diabetic foot risk stratification system
Need for a change	-Lack of referral pathway from the general practitioner to the private podiatrist -Reimbursement for podiatry consultations in Belgium

Inclusion criteria for this research were registered podiatrists working part-time or full-time in the private sector in Flanders, Belgium. Therefore, podiatrists working solely in multidisciplinary diabetic foot clinics in the public sector (MDFCs) were excluded from the study, as these clinics are bound to guidelines for clinical audits. Other health care professionals such as diabetes nurses, physiotherapists and GPs were also excluded from the study. The survey was only available in English and Dutch. As a result, the link to the survey was not sent out to podiatrists working in the private sector in the French part of Belgium, Wallonie. These podiatrists were therefore also excluded from the study.

### Data analysis

Data analysis was conducted solely by the researcher. Data from the survey were kept within the “JISC online survey” tool and analyzed using Excel (Microsoft 365) and SPSS Statistics version 26 for Windows (IBM Corporation). Categorical data were compared using a Fishers exact test. A p-value of < 0.05 was considered statistically significant. Data from free text responses to the survey questions and online interviews were coded using the inductive thematic analysis described by Braun and Clarke [15]. Personal details of the interviewees were removed from the interview transcripts. Thematic analysis of the qualitative data involved familiarization and analysis of data, developing core themes around podiatrists’ experiences and opinions and reviewing these themes before reporting the results. The core themes and most interesting quotes were translated in English after the thematic analysis. All interview transcripts were returned to the participants for comments and/or corrections. As a result, member checking along with the methodological triangulation, using survey and interviews for data collection, helped to mitigate bias in coding.

## Results

### Survey

Out of the 362 podiatrists contacted in Flanders, Belgium, a total of 50 participated in the survey (14% response rate). Demographic data from the respondents are presented in Table 2.

**Table 2:** Demographics of respondents

Duration of employment as a podiatrist ^a^:	(*N* = 50)
•0–5 years	50% (25)
•5–10 years	16% (8)
• > 10 years	34% (17)
Practice office setting of podiatrists:	(*N* = 50)
•Private practice	40% (20)
•Part-time hospital/ Part-time private (multidisciplinary) practice	32% (16)
•Multidisciplinary private practice	28% (14)
Number of diabetic patients treated in an average week ^a^:	(*N* = 50)
•Less than 5 patients	38% (19)
•Between 5 and 10 patients	24% (12)
•More than 10 patients per week	38% (19)

^a^Variables were not mutually exclusive. Results should be interpreted with caution

#### Diabetic foot assessment methods

**Fig. 1:**
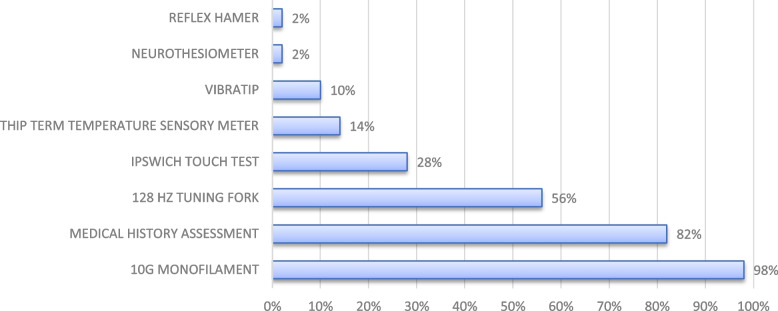
Screening tests for diabetic neuropathy. Detailed summary of tests used to assess diabetic neuropathy in private podiatry practices in Flanders, Belgium. *N* = 50 podiatrists

**Fig. 2:**
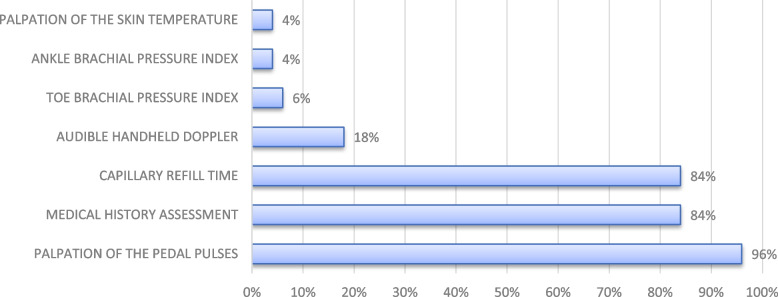
Screening tests for peripheral arterial disease. Detailed summary of tests used to perform the vascular assessment of the diabetic foot in private podiatry practices in Flanders, Belgium. *N* = 50 podiatrists

The survey investigated which screening assessment methods were routinely performed to identify diabetic neuropathy and periperal arterial disease (PAD). These results are presented in Fig. 1 and 2. The results of the survey indicate that the most reported screening tests for diabetic neuropathy are history of symptoms of neuropathy (82%) and the 10 g monofilament test (98%) (see Fig. 1). In addition to history and 10 g monofilament test, at least one other test was used by 72% of the respondents such as the 128 Hz tuning fork or the Ipswich Touch test [16].

For the assessment of PAD, a minimum combination of medical history, palpation of the pedal pulses and capillary refill time was used by 68% of the respondents. An audible handheld doppler, ankle brachial pressure index (ABPI) and toe brachial pressure index (TBPI) were seldom used (18%, 4% and 6% respectively). However, when podiatrists were asked which tests they are not currently performing but would like to, 22% reported they would like to use an audible handheld doppler and 6% would like to perform an ABPI.

The survey investigated the use of complementary tests for the diabetic foot assessment. Inspection of foot deformities, footwear (shoes and socks) and skin/nails/wounds was reported by 6%, 8% and 18% of the podiatrists respectively. Clinical tests to detect joint mobility was the most reported complementary test (28%).

#### Diabetic foot assessment guidelines or guidance documents and risk stratification systems

Sixty-six percent of all podiatrists use guidance documents or guidelines for the assessment of the diabetic foot. The Fisher’s exact test was used to determine the relationship between years of experience and the use of guidelines or guidance document for diabetic foot assessments. Although recently graduated podiatrists are introduced to the latest guidelines of the IWGDF, the test showed that they are not more likely to use guidelines compared to experienced podiatrists (fisher exact test. *p* = 0,837). The findings of this survey indicate that a minimum of 9 different guidance documents or guidelines are used by private podiatrists working in Flanders, Belgium. Most of the podiatrists (24%) develop their own guidance documents or guidelines for the diabetic foot assessment. 21% of the respondents did not specify the name of the document used for this assessment while others reported published risk guidance classification documents such as the guidance documents available on patient management software designed for podiatrists (12%), the Sims classification (9%) [17] or the perfusion, extent, depth, infection and sensation classification system (9%) [18] (PEDIS), the IWGDF guidelines (6%) [14], documents retrieved from MDFC (9%), DN4 and VAS-score questionnaire (6%) and documents retrieved from the podiatry undergraduate course (3%).

Risk stratification systems are used to determine the risk of developing a DFU and are often used to determine the frequency of podiatry visits. However, only 66% of the respondents use a diabetic foot risk stratification system. The most popular system (42%) is the one provided by the NIHDI [19] to determine the reimbursement of podiatry consultations for patients with diabetes. The “SIMS classification” [17] is the second most described risk stratification system in use (36%).

#### Frequency of diabetic foot assessments

**Fig. 3:**
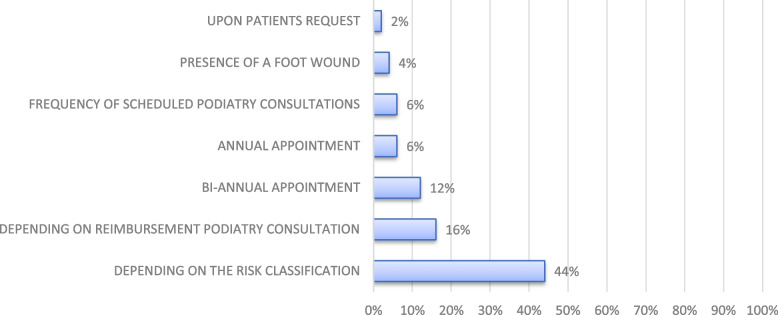
Frequency of diabetic foot assessments. How often and when do podiatrists perform a diabetic foot assessment in private podiatry practices in Flanders, Belgium. *N* = 50 podiatrists

Figure 3 shows how podiatrists determine the frequency of diabetic foot assessments. It highlights the two most commonly cited reasons for assessment frequency being the risk classification of the patient (44%) and depending on the reimbursement arrangements for podiatry consultations (16%). Other reported reasons were the frequency of scheduled appointments (6%), presence of a foot wound (4%) or patients request for a diabetic foot assessment (2%). Some podiatrists perform the diabetic foot assessment only during annual (6%) or bi annual (12%) podiatry consultations.

### Interviews

#### Guidance documents or guidelines for the diabetic foot assessment

The majority of the participants interviewed reported the use of a variety of guidelines for the diabetic foot assessment in private practices, which was also apparent in the survey results. Furthermore, these guidelines and guidance documents were retrieved in various ways.

“* I attend the International Symposium on the Diabetic Foot every year”*

*“ We retrieved guidelines or guidance documents through conferences, guidelines from other countries and following Prof. Dr. Armstrong on social media”*

*“ I use the guidance document that I received during my undergraduate internship”*

When asked about possibly introducing a national diabetic foot assessment guideline, the participants suggested that it would be impossible to introduce a national guideline immediately. They reported that the process of developing and introducing a national guideline should start with increasing the Belgian private podiatrists’ awareness of the IWGDF guidelines.

*“Communicating the latest international standards on diabetic foot assessment, through BVP-ABP (the podiatric medical association in Belgium), would be a step in the right direction. Although, we do not know if podiatrists will read this information.”*

#### Diabetic foot assessment

It was apparent in the survey results that podiatrists use a minimum combination of medical history, palpation of the pedal pulses and capillary refill for the vascular assessment. The interviews explored why highly recommended methods such as a Doppler or ABPI are not integrated in the diabetic foot assessment in private podiatry practices in Belgium.

The majority of participants agreed with the statement that it is not worth investing in the costly equipment for ABPI or Doppler.

*“You have to buy additional equipment and I am wondering what is the cost–benefit analysis?”*

*“It is quite expensive and you do not need it that often. I would rather invest in other equipment that I need for daily use”.*

This view was echoed by another participant who stated that besides this costly investment, the current pricing of podiatry consultations and non-existent referral pathways for patients with diabetes hold podiatrists back to invest in the equipment needed.

*“The pricing of the podiatry consultations has not been correctly determined. I really would like to invest in this equipment but if I only need to use it once a year due to the lack of referral of patients with diabetes, it is not worth the investment.*

Another common view amongst interviewees was the inconsistencies in the interpretation of the diabetic foot risk factor between different private podiatrists. It was suggested that although there is no national guideline on diabetic foot assessment, podiatrists use the same assessment methods. However, when the results of this assessment must be interpreted there could be inconsistencies in how these are used to stratify the patients risk factor for developing a DFU.

*“Patient with a medical history of revascularization, presenting with good palpable pedal pulses remains a high risk foot in my opinion. However, colleagues could interpret this differently. They could reason that because the pedal pulses are palpable the patient would fall back to the low risk category.”*

#### Need for change

A recurrent theme in the interviews was a sense amongst interviewees that some issues in assessing the diabetic foot highlight the need for change in the diabetic foot care. Firstly, there is no established referral pathway from the GP to the private podiatrist in Belgium.

*“There are few GPs referring patients with diabetes for a diabetic foot assessment. I have been trying for years to change this, without any result. They only refer patients when DFUs occur.”*

Secondly, the lack of referral could be attributed to the fact that GPs are not aware of the professional capabilities of podiatrists.

*“I think GPs have no idea of what the podiatrists’ professional capabilities really are. They suppose we solely perform diabetic foot care and do not realize we perform a thorough diabetic foot assessment prior to the foot care. What does the GP expect from the podiatrist and vice versa? I think this needs further discussion.”*

Lastly, podiatry consultations are only reimbursed twice a year, which is not sufficient for patients with a moderate to high risk of developing a DFU.

*“A lot of high risk patients need frequent podiatry consultations. However, they also have a lot of other medical expenses, so they stick to the two reimbursed podiatry consultations a year because they can’t afford it. If we could treat these patients every month, this would reduce the occurrence of diabetic foot problems remarkably.”*

*“Patients with diabetes have a lot of medical expenses. Podiatry consultations cost between 30 to 35 euros. If you have to pay this out of your own pocket every time, these medical expenses will increase and some patients will eventually cut out these expenses.”*

## Discussion

To our knowledge, this is the first exploratory mixed method study that evaluated the podiatrists’ experiences and methods in assessing the diabetic foot in the private sector in Flanders, Belgium. The major findings of this research were firstly the lack of use of non-invasive tests for the vascular assessment of the diabetic foot. Secondly, only 66% of the respondents use guidelines to assess the DF. Moreover, this research has shown that the IWGDF guidelines have not yet been implemented in the private podiatry practices in Flanders, Belgium. Lastly, one third of the respondents do not use a risk stratification system to identify patients at risk for developing a DFU.

The most important finding was the limitation of non-invasive tests used for the vascular assessment of the diabetic foot. PAD is an independent risk factor for subsequent DFU [20]. Studies have shown that it is present in up to 50% of patients presenting with a DFU [21, 22]. Moreover, previous research has established that patients with diabetes with PAD were five times more likely to have undergone a lower-extremity amputation (LEA) and had higher mortality compared to non-diabetes patients [23–25]. The UK NICE guidelines recommend to assess the vascular status as an important predictor of ulceration in the diabetic foot [26]. Considering this evidence, it seems that identification of PAD in patients with diabetes is key in minimizing the risk of LEA. The results of the survey and interviews showed that the vascular assessment in private podiatry practices in Flanders, Belgium solely exists of a medical history and palpation of the pedal pulses. These results are consistent with previous research, which evaluated the vascular assessment techniques of podiatrists in the UK [27]. Nevertheless, the IWGDF guidelines suggest that the presence of palpable foot pulses cannot be used in isolation to reliably exclude PAD [28]. Pedal pulse examination has a poor sensitivity and is not independently sufficient to conclusively diagnose PAD [29, 30]. Therefore, guidelines recommend a more objective evaluation with a Doppler, ABPI or TBPI for identifying PAD [28–30]. Research has shown that non-invasive testing such as Doppler and TBPI are more accurate and viable screening tests to identify PAD among patients with diabetes [31–33]. TBPI is often preferred for diagnostic testing because research has shown that this test provides a more accurate diagnosis in patients with diabetes with carotid atherosclerosis compared to ABPI [32, 34, 35]. The results of the interviews of this study indicated that the lack of podiatry consultation reimbursement and referral pathways hinders private podiatrists in Flanders, Belgium to invest in the equipment needed for noninvasive testing. Lack of equipment has been reported as a frequent barrier to performing a vascular assessment in previous studies performed in the UK and Australia [36, 37]. This shows that in order to improve the quality of diabetic foot assessments, podiatrists should get the opportunity to invest in proper equipment.

The second finding of this research was that the IWGDF guidelines have not yet been implemented in private podiatry practices in Flanders, Belgium. Since 1999, the IWGDF has developed international clinical practice guidelines for the prevention and management of the diabetic foot [14]. These guidelines are systematically developed statements to assist health care professionals’ decisions, to standardize the diabetic foot care and improve the quality of health care [38, 39]. The IWGDF advises that those guidelines may have to be adapted based on local circumstances taking into account accessibility to health care resources and various cultural factors [14]. When nations consider to develop a national diabetic foot assessment guideline, it is advised to adopt a similar methodology to that used by the IWGDF [40, 41]. Moreover, the guidelines must be as specific as possible to reduce ambiguity and confusion among clinicians managing patients with DFUs [40]. Several studies have shown that developing a national diabetic foot assessment guideline, based on the international recommendations, not only increases the frequency of diabetic foot assessments [42, 43] but also reduces the incidence of diabetes-related LEA [19, 44]. However, in Belgium, the international recommendations have not yet been implemented into a national diabetic foot assessment guideline. This could be the reason why only 66% of all private podiatrists in Flanders, Belgium reported to use guidance documents or guidelines for the assessment of the diabetic foot. Solely 6% of these podiatrists are using the IWGDF guidelines. It also raises a question to what are the other 34% using? Although, with only a response rate of 14%, we do not have a true representative sample of private podiatrists in Flanders, Belgium and these results must therefore be interpreted with caution.

Another possible explanation for the lack of implementation of diabetic foot assessment guidelines in private podiatry practices in Flanders, Belgium could be the variety of available guidelines or guidance documents published by various organizations and experts in the field [45, 46]. This could create confusion among podiatrists as to which guidelines should be implemented in clinical practice and explains why 9 different guidelines or guidance documents were identified as being used in private podiatry practices in Flanders, Belgium. Moreover, studies have shown that there is a high variability in the recommended methods for the diabetic foot assessment and a lack of consistency regarding the levels of evidence and grades of these recommended methods between different guidelines [45, 47, 48]. As a result, the variation of guidelines used in private podiatry practice in Flanders, Belgium could lead to differences in interpretation of the diabetic foot risk stratification system between podiatrists and affect the quality of diabetic foot care ultimately received by the patient.

The concerns regarding the inconsistent interpretation of the diabetic foot risk stratification system among the private podiatrists were widespread in the interviews. This concern could be explained by the lack of implementation of these systems in podiatry practice, which was apparent in the survey results. One third of the respondents do not use a risk stratification system to identify patients at risk for developing a DFU. Diabetic foot risk stratification systems are designed to determine the appropriate management and assessment frequency of the diabetic foot [46]. Studies of Boyko et al. [49] and Leese et al. [50] showed that risk stratification systems based on the IWGDF guidelines have an excellent ability in accurately quantifying and defining an individual’s risk of developing a DFU. Moreover, it provides a more accurate prediction of the foot ulcer risk than the individual results considered in isolation [49]. The survey results indicated that one-third of the podiatrists are not using any risk stratification system. As a result, it could be assumed that these podiatrists determine the patients risk factor based on individual predictors potentially resulting in inconsistent diabetic foot risk scores. Podiatrists that did use a risk stratification system, most frequently rely on the system provided by the Belgian NIHDI [51] (Table 3) which is based on the on the Coleman’s risk stratification [52, 53]. Although this system is provided by the Belgian Institute, it is important to note that this risk stratification has never been adapted to the latest research or recommendations of the IWGDF. Moreover, there are no studies that have validated this risk stratification system, which could explain why there could be inconsistencies in the diabetic foot risk stratification interpretation between podiatrists. Therefore, adopting a new risk stratification system in compliance with the latest international recommendations could decrease the inconsistencies in the interpretation of the diabetic foot risk stratification between podiatrists. This would ultimately improve the diabetic foot care for patients at risk for developing a DFU.

**Table 3:** Risk stratification system NIDHI & IWGDF

Risk Group	Risk classification Belgian NIDHI [40]	IWGDF guidelines [9]
0	-	no LOPS or PAD
1	LOPS	LOPS or PAD
2	A)Moderate foot deformities such as prominence of metatarsal heads, hyperkeratosis and/or flexible hammer- or claw toes and/or moderate hallux abducto valgus (< 30°) B)Severe foot deformities	LOPS + PAD LOPS + foot deformity PAD + foot deformity
3	PAD History of DFU Amputation Charcot	LOPS or PAD and one or more of the following: •History of foot ulcer •LEA (minor or major) •End-stage renal disease

*LOPS* Loss of protective sensation, *PAD* Peripheral arterial disease, *DFU* Diabetic foot ulcer, *LEA* Lower extremity amputation

### Limitations

The present study has presented a number of limitations and the results should be interpreted cautiously. Firstly, the trustworthiness of this study was subject to certain limitations. The transferability of this research was affected by the small sample size. Invitations for the survey and interviews were solely sent out to private podiatrists in Flanders, Belgium. Moreover, only a small proportion of private podiatrists responded (14%) resulting in a poor external validity. Moreover, this study was conducted as an exploratory mixed method research. Therefore, there was no pilot study or peer review performed prior to this research to validate the survey and interview questions. This resulted in a poor reliability and dependability. In order to generalize the results nationally, this study should be repeated including podiatrists working in the private sector in Wallonie and measures should be undertaken to improve the response rate and to validate the survey and interview questions.

Secondly, the survey limited the researcher in her ability to further explore the content of the different risk stratification systems used in private podiatry practice. The second most reported risk stratification used was the “SIMS risk stratification system”. This system was developed in 1988 [17] and introduced in the neighboring country the Netherlands. According to diabetic foot assessment guidelines in the Netherlands, their risk stratification system has been adapted in 2006 to the current IWGDF guidelines. However, the term “SIMS” was kept as podiatrists kept associating the term with diabetic foot risk stratification [54]. As a result it is possible that Belgian private podiatrists reporting the use of the “SIMS risk stratification system” actually use the current IWGDF guidelines, not the original reported system of 1988, which could have influenced the results related to this survey question.

Lastly, the audit reports from the MFCS in secondary care in Belgium provide the only data available on national diabetic foot care. The lack of organization of services for the diabetic foot in the private sector results in a gap of knowledge on the current diabetic foot assessment methods and foot care. Furthermore, it makes it impossible to analyze how the current practice could influence the results of the audit report from the MFCS.

## Conclusion

The findings of this study demonstrated that podiatrists in the private sector in Flanders, Belgium rarely use non-invasive tests such as the audible handheld doppler, ABPI or TBPI for the vascular assessment of the diabetic foot. Diabetic foot assessment guidelines and risk stratification system to identify patients at risk for developing a DFU are not frequently used in private podiatry practices. Furthermore, the international guidelines of the IWGDF have not yet been implemented in these practices, which highlights the need for the development of a uniform national diabetic foot assessment guideline.

These findings are important targets for further investigation. In order to generalize these results, future research targeting registered podiatrists working in the private and public sectors in Belgium is needed. Conducting a pilot study or peer review to validate the questionnaire could improve the reliability of these future studies.

## Data Availability

The datasets used and/or analysed during the current study are available from the corresponding author on reasonable request.

## Abbreviations

ABPI: Ankle brachial pressure index
DF: Diabetic foot
DFU: Diabetic foot ulceration
DM: Diabetes Mellitus
GP: General practitioner
IWGDF: International Working Group of the Diabetic Foot
LEA: Lower extremity amputation
LOPS: Loss of protective sensation
MDFC: Multidisciplinary Foot Clinic
MFCS: Multidisciplinary foot care services
NIHDI: National Institute for Health and Disability Insurance
PAD: Peripheral arterial disease
UK: United Kingdom
